# Isorhynchophylline, a Potent Plant Alkaloid, Induces Apoptotic and Anti-Metastatic Effects in Human Hepatocellular Carcinoma Cells through the Modulation of Diverse Cell Signaling Cascades

**DOI:** 10.3390/ijms18051095

**Published:** 2017-05-19

**Authors:** Hanwool Lee, Seung Ho Baek, Jong Hyun Lee, Chulwon Kim, Jeong-Hyeon Ko, Seok-Geun Lee, Arunachalam Chinnathambi, Sulaiman Ali Alharbi, Woong Mo Yang, Jae-Young Um, Gautam Sethi, Kwang Seok Ahn

**Affiliations:** 1 College of Korean Medicine, Kyung Hee University, 24 Kyungheedae-ro, Dongdaemun-gu, Seoul 02447, Korea; okhwko@naver.com (H.L.); baeksh@woosuk.ac.kr (S.H.B.); 88milkyway@hanmail.net (J.H.L.); sunny10526@nate.com (C.K.); gokjh1647@gmail.com (J.-H.K.); seokgeun@khu.ac.kr (S.-G.L.); wmyang@khu.ac.kr (W.M.Y.); jyum@khu.ac.kr (J.-Y.U.); 2 College of Korean Medicine, Woosuk University, 46 Eoeun-ro, Wansan-gu, Jeonju-si, Jeollabuk-do 54987, Korea; 3 Department of Botany and Microbiology, College of Science, King Saud University, Riyadh 11451, Saudi Arabia; dr.arunmicro@gmail.com (A.C.); sharbi@ksu.edu.sa (S.A.A.); 4 School of Biomedical Sciences, Curtin Health Innovation Research Institute, Curtin University, Perth, WA 6009, Australia; 5 Department of Pharmacology, Yong Loo Lin School of Medicine, National University of Singapore, Singapore 117600, Singapore

**Keywords:** isorhynchophylline, apoptosis, invasion, migration, cell signaling

## Abstract

Isorhynchophylline (Rhy) is an active pharmacological component of *Uncaria rhynchophylla* that has been reported previously to exert significant antihypertensive and neuroprotective effects. However, very little is known about its potential anti-cancer activities. This study was carried out to evaluate the anticancer effects of Rhy against various human carcinoma cell lines. We found that Rhy exhibited substantial cytotoxic effect against human hepatocellular carcinoma HepG2 cells when compared with other human carcinoma cell lines including those of lung, pancreas, prostate, head and neck, breast, multiple myeloma, brain and renal cell carcinoma. Rhy induced apoptosis as characterized by accumulation of cells in sub G1 phase; positive Annexin V binding; activation of caspase-8, -9, and -3; and cleavage of PARP (poly-ADP ribose polymerase). This effect of Rhy correlated with the down-regulation of various proteins that mediated cell proliferation, cell survival, metastasis, and angiogenesis. Moreover, cell proliferation, migration, and constitutive CXCR4 (C-X-C chemokine receptor type 4), MMP-9 (Matrix metallopeptidase-9), and MMP-2 expression were inhibited upon Rhy treatment. We further investigated the effect of Rhy on the oncogenic cell signaling cascades through phospho-kinase array profiling assay. Rhy was found to abrogate phospho-p38, ERK, JNK, CREB, c-Jun, Akt, and STAT3 signals, but interestingly enhanced phospho-p53 signal. Overall, our results indicate, for the first time, that Rhy could exert anticancer and anti-metastatic effects through regulation of multiple signaling cascades in hepatocellular carcinoma cells.

## 1. Introduction

Isorhynchophylline (Rhy) is one of the major oxindole alkaloids isolated from *Uncaria rhynchophylla*, which has already been extensively used for the treatment of asthma, cancer, cirrhosis, diabetes, hypertension, stroke and rheumatism in tropical regions, such as Southeast Asia, Africa and southeast America [1]. Previous studies have demonstrated that Rhy mainly acts on cardiovascular and central nervous system diseases including hypertension, brachycardia, arrhythmia, sedation, vascular dementia, and amnesia [2]. Recently, Kaiser et al. have elegantly evaluated the potential effect of pentacyclic oxindole alkaloids isolated from *Uncaria tomentosa* on genotoxicity and cytotoxicity against human leukocytes, human bladder cancer cell line (T24) and human glioblastoma cell line (U-251-MG) and found that diverse chemotypes exhibited differential selectivity against human malignant cells [3]. Rhy has also been reported to exhibit anti-inflammatory activities in mouse microglial cells [4,5]. However, no reports have been published so far on the anticancer potential of Rhy, and possible molecular mechanism(s) underlying its anticancer effects.

Natural products play an important role in the process of anticancer drug discovery. Because of pharmacological safety, plant-derived natural products as well as their semisynthetic and synthetic analogs contribute significantly to the process of development of novel anti-neoplastic agents [6,7]. For a long time, deregulation in the process of apoptosis has been a significant cause of carcinogenesis [8]. Now it is generally agreed that during the formation of cancer the suppression of apoptotic signals could have a very significant effect [9]. Triggering apoptosis in cancer cells has thus become an important method of enhancing the results of therapy during the treatment of cancer. Much evidence has demonstrated that several phytochemicals exert anti-tumorigenic activities by several processes, including preventing the activation of pro-carcinogens, inhibiting cell proliferation, invasion, and angiogenesis, and stimulating sustained apoptosis in tumor cells [10]. A number of dietary agents derived from natural sources can also regulate mitochondrial biogenesis and also simultaneously target various signaling molecules implicated in the apoptotic pathway [11,12]. For example, triptolide, a major active ingredient extracted from the widely used Chinese herb *Tripterygium wilfordii* Hook f. that has been extensively analyzed for its anticancer effects was reported to induce pathological changes of heart tissue and exhibit cardiotoxicity through the modulation of the mitochondria-mediated apoptotic signaling pathway [13].

The purpose of this study was to investigate the potential anticancer effects of Rhy and elucidate its underlying molecular mechanisms. We particularly aimed to determine the effect of Rhy on the induction of apoptosis and inhibition of metastatic activity in tumor cells. In our experiments, Rhy was found to substantially downregulate the expression of several anti-apoptotic, proliferative, metastatic, and angiogenic gene products, leading to the induction of apoptosis through caspase-8, -9, and -3 activation, and also inhibited migratory and invasive potential of tumor cells.

## 2. Results

### 2.1. Rhy Suppressed the Cell Viability in Variety of Tumor Cells

**Figure 1:**
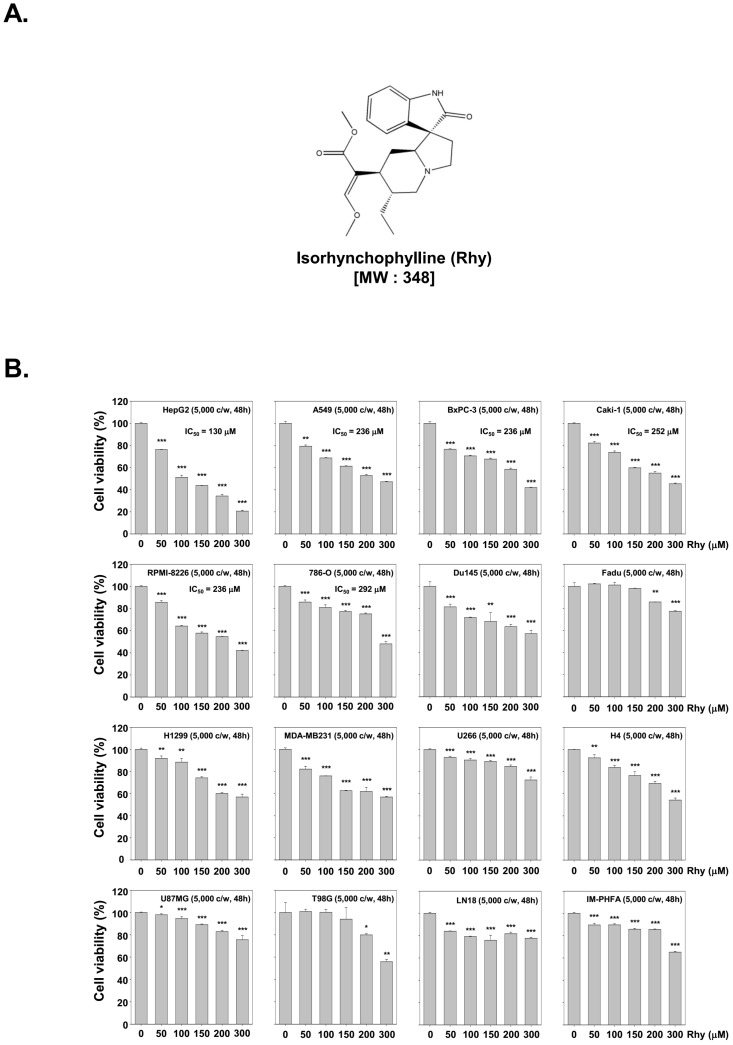
Cytotoxicity of isorhynchophylline (Rhy) on various cell lines: (**A**) Structure of Rhy; and (**B**) HepG2, A549, BxPC-3, Caki-1, RPMI-8226, 786-O, Du145, FaDu, H1299, MDA-MB-231, U266, H4, U87MG, T98G, LN18, and IM-PHFA cells were treated with Rhy (0, 50, 100, 150, 200, or 300 µM) for 48 h. Values represent the mean ± SD of triplicate cultures (* *p* < 0.05, ** *p* < 0.01, *** *p* < 0.001). Cell viability was analyzed by the MTT method as described under Materials and Methods.

The structure of Rhy has been shown in Figure 1A. To examine the anti-tumor activity of Rhy, HepG2, A549, BxPC-3, Caki-1, RPMI-8226, 786-O, Du145, FaDu, H1299, MDA-MB-231, U266, H4, U87MG, T98G, LN18 and IM-PHFA cells were treated with Rhy (0, 50, 100, 150, 200, or 300 µM) for 48 h, and then cell viability was measured by MTT assay. As shown in Figure 1B, Rhy exhibited greatest cytotoxicity against HepG2 cells as compared to other tumor cells as well as immortalized primary human fetal astrocytes (IM-PHFA).

### 2.2. Rhy Repressed the Expression of Various Proteins Involved in Anti-Apoptosis, Proliferation, Metastasis and Angiogenesis

**Figure 2:**
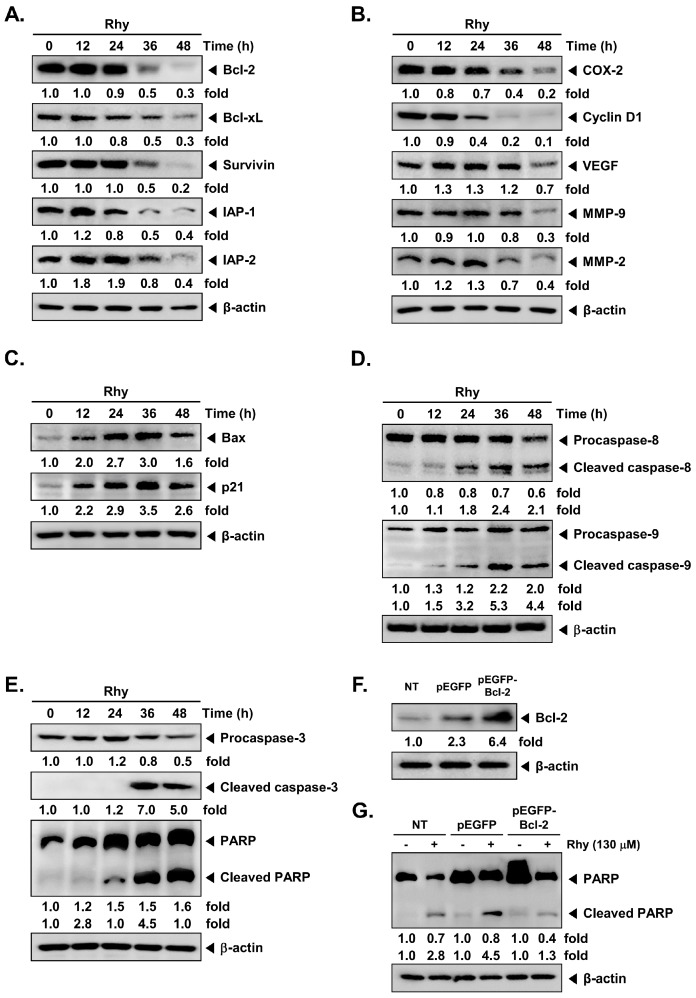
Induction of apoptosis by Rhy in HepG2 cells. (**A**–**C**) Rhy modulates expression of various proteins involved in anti-apoptosis, proliferation, angiogenesis, metastasis, and pro-apoptosis. HepG2 cells (1 × 10^6^ cells/well) were incubated with Rhy (130 µM) for 0, 12, 24, 36, and 48 h. Whole-cell extracts were prepared, and 20 µg of the whole-cell lysate was resolved by sodium dodecyl sulfate polyacrylamide gel electrophoresis (SDS-PAGE), electrotransferred to nitrocellulose membrane, sliced from the membrane based on the molecular weight, and then probed with antibodies against Bcl-2, Bcl-xL, survivin, inhibitors of apoptosis protein (IAP)-1/2, COX-2, Cyclin D1, VEGF, MMP-9/2, Bax, and p21 as described in the Materials and Methods. The same blots were stripped and reprobed with β-actin antibody to verify equal protein loading; (**D**,**E**) HepG2 cells (1 × 10^6^ cells/well) were treated with Rhy (130 µM) for 0, 12, 14, 36, and 48 h. Thereafter, equal amounts of lysates were analyzed by Western blotting analysis using antibodies against caspase-8/9/3 and PARP. The same blots were stripped and reprobed with β-actin antibody to verify equal protein loading; (**F**) Cells were transiently transfected with pEGFP-Bcl-2 or pEGFP (control vector) plasmid. Bcl-2 protein was overexpressed in pEGFP-Bcl-2 transfected HepG2 cells compared with control. The results shown are representative of the three independent experiments; (**G**) Transiently transfected cells were treated with Rhy for 48 h. Thereafter, equal amounts of lysate were analyzed by Western blot analysis using antibodies against PARP and β-actin.

We next examined the effects of Rhy on the expression of various proteins involved in anti-apoptosis, proliferation, metastasis and angiogenesis in HepG2 cells. As depicted in Figure 2A Rhy suppressed the expression of anti-apoptotic gene products such as Bcl-2 (B-cell lymphoma-2), Bcl-xL (B-cell lymphoma-extra large), Survivin, IAP-1 (inhibitors of apoptosis-1) and IAP-2 (inhibitors of apoptosis-2) in a time-dependent manner. Figure 2B shows that Rhy also substantially repressed the expression of proteins linked with metastasis and angiogenesis including COX-2 (cyclooxygenase-2), VEGF (vascular endothelial growth factor), MMP-9, and MMP-2 and cell cycle regulatory protein such as Cyclin D1.

### 2.3. Rhy Induced the Expression of Bax and p21 Proteins

The Bcl-2 family proteins have emerged as critical regulators of the mitochondria mediated apoptosis by functioning as either promoters (e.g., Bax (Bcl-2-associated X) and Bak (Bcl-2 homologous antagonist/killer)) or inhibitors (e.g., Bcl-2 and Bcl-xL) of the cell death process [14]. Once activated, Bax permeabilizes the mitochondrial outer membrane, resulting in the release of cytochrome c and other pro-apoptotic factors that can induce caspase activation and programmed cell death [15]. Besides, the cyclin-dependent kinase (CDK) inhibitor p21 is a prototypical member of the CIP/KIP family of CDK inhibitors. It negatively modulates cell cycle progression by inhibiting the activities of cyclin D/CDK4 complexes and can block DNA replication by binding to proliferating cell nuclear antigen [16]. We found that Rhy induced the expression of Bax and p21 proteins in a time-dependent manner in HepG2 cells (Figure 2C).

### 2.4. Rhy Activated Caspase-3 and Caused PARP Cleavage

We also evaluated the effect of Rhy on the apoptotic caspase-PARP axis in HepG2 cells. Rhy induced the cleavage of procaspase-8 and procaspase-9 as seen by the appearance of its cleavage products (Figure 2D). As shown in Figure 2E, Rhy also induced a time-dependent activation of caspase-3. Activation of caspase-3 led to the cleavage of a 116 kDa PARP (poly-ADP ribose polymerase) protein into 87 kDa fragments. These results clearly suggest that Rhy can induce caspase-3-dependent apoptosis in HepG2 cells.

### 2.5. Overexpression of Bcl-2 Attenuated Rhy-Mediated Apoptosis

We next investigated whether overexpression of Bcl-2 by pEGFP-Bcl-2 plasmid can prevent the observed effects of Rhy on apoptosis. The cells transfected with pEGFP-Bcl-2 clearly showed overexpression of Bcl-2 as compared with those transfected with only control plasmid (Figure 2F). The same blots were stripped and reprobed with β-actin antibody to verify equal protein loading. As shown in Figure 2G, overexpression of Bcl-2 led to the attenuation of Rhy-mediated cleavage of PARP as compared with the control cells.

### 2.6. Rhy Caused the Accumulation of the Cells in the Sub-G1 Phase

**Figure 3:**
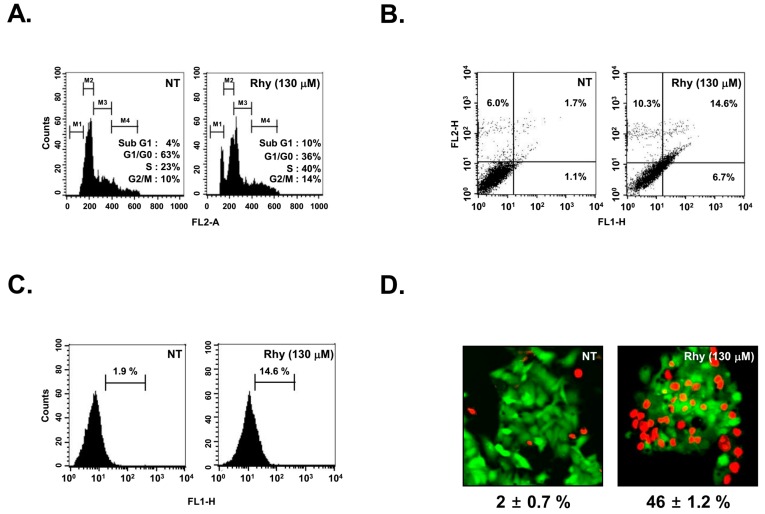

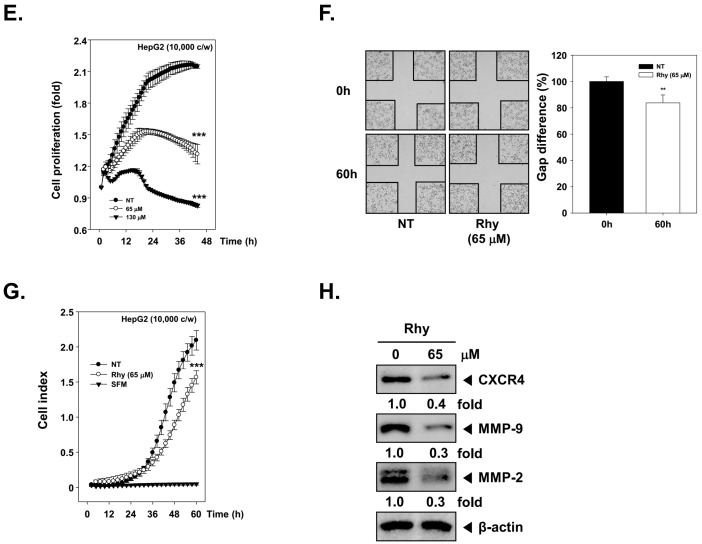
Apoptotic and anti-metastatic effect of Rhy in HepG2 cells. (**A**) After HepG2 cells (1 × 10^6^ cells/well) were seeded onto six-well plates, they were treated with 130 µM of Rhy for 36 h. Then, the cells were harvested, washed with a cold PBS buffer, and digested with RNase. Cellular DNA staining with PI and flow cytometric analysis was done to determine the cell cycle distribution as described in the Materials and Methods; (**B**) HepG2 cells (1 × 10^6^ cells/well) were treated with Rhy for 48 h. The cells were incubated with an FITC-conjugated Annexin V antibody and then analyzed by a flow cytometry as described in Materials and Methods. Data are representative of the best quality data collected from three individual experiments with similar results and the percentage of late apoptotic cells (upper right quadrant); (**C**) After HepG2 cells (1 × 10^6^ cells/well) were seeded onto six-well plates, they were treated with 130 µM of Rhy for 48 h. The cells were fixed and incubated using TUNEL reaction solution and then analyzed by a flow cytometry. Data are representative of the best quality data collected from three individual experiments with similar results; (**D**) HepG2 cells were treated with Rhy (130 µM) for 36 h. Cells were stained with a live/dead assay reagent for 30 min and then analyzed under a fluorescence microscope (40×) as described in Materials and Methods. Percentage of apoptosis is indicated in the inset; (**E**) HepG2 cells were plated in triplicate and treated with Rhy (65 or 130 µM), then cell proliferation was measured by real time cell analysis (RTCA). Values represent the mean ± SD of triplicate cultures (*** *p* <0.001); (**F**) Wound healing assay was performed for evaluating the inhibitory effect of Rhy on HepG2 cells migration. Confluent monolayers of HepG2 cells were scarred, and repair was monitored microscopically after 60 h of treatment with 65 µM Rhy. Width of wound was measured at time 0 and 60 h of incubation with and without Rhy. Values represent the mean ± SD of triplicate cultures (** *p* < 0.01); (**G**) Invasion assay was performed using the Roche xCELLigence Real-Time Cell Analyzer (RTCA) DP instrument (Roche Diagnostics GmbH, Mannheim, Germany) as described in Materials and Methods. We tested HepG2 cells invasion activity (40,000 cells/well) in the matrigel-coated CIM (cellular invasion/migration)-Plate 16 with Rhy (65 µM); (**H**) Rhy downregulated expression of CXCR4 and MMP-9/2. HepG2 cells (1 × 10^6^ cells/well) were incubated with Rhy (65 µM) for 60 h. Whole-cell extracts were prepared, and 20 µg of the whole-cell lysate was resolved by sodium dodecyl sulfate polyacrylamide gel electrophoresis (SDS-PAGE), electrotransferred to nitrocellulose membrane, sliced from the membrane based on the molecular weight, and then probed with antibodies against CXCR4, MMP-9, and MMP-2 as described in Materials and Methods. The same blots were stripped and reprobed with β-actin antibody to verify equal protein loading. FL2-A, gating for single cells based on the area; FL1-H, relative intensity of GFP florescence; NT, Non treat.

We determined the effect of Rhy on cell cycle distribution in HepG2 cells. Figure 3A shows that Rhy increased the cell accumulation in the sub-G1 phase, which is indicative of apoptosis as compared with the non-treated (NT) cells (Figure 3A).

### 2.7. Rhy Promoted Substantial Apoptotic Cell Death

We next examined the effect of Rhy on cellular apoptosis in HepG2 cells. We found that Rhy increased early apoptotic cells in HepG2 cells as observed by the annexin V assay that increased up to 14.6% as compared with the non-treated cells (1.7%) (Figure 3B). The induction of apoptosis was further confirmed by TUNEL staining assay. A significant number (up to 14% of HepG2 cells) stained positively for TUNEL in Rhy treated group as compared with the NT cells (1.9%) (Figure 3C). The Live and Dead assay (which measures intracellular esterase activity and plasma membrane integrity) shows that treatment of Rhy increases population of apoptotic cells from 2% to 46% (Figure 3D)

### 2.8. Rhy Suppressed the Proliferative Activity of HepG2 Cells

To specifically examine the anti-tumor activity of Rhy in HepG2 cells, the cells were treated with Rhy (65 and 130 µM) for indicated time intervals, and then the cell proliferation was analyzed using a real time cell analyzer (RTCA; Roche). As shown in Figure 3E, Rhy suppressed the proliferation in HepG2 cells in a dose-dependent manner. 

### 2.9. Rhy Suppressed HepG2 Cells Migration and Invasion

The effect of Rhy on HepG2 cells migration was elucidated, and it was found, using an in vitro wound healing assay, that HepG2 cells migrated slower under the influence of Rhy (Figure 3F). Whether Rhy can modulate HepG2 cells invasion activity was also investigated. To determine this, cells were seeded to the Matrigel (BD Biosciences, San Diego, CA, USA)-coated CIM-Plate 16 with or without Rhy and examined for invasion. As shown in Figure 3G, Rhy significantly suppressed tumor cells invasion activity. We also evaluated the effect of Rhy on the expression of CXCR4, MMP-9, and MMP-2, proteins which play a critical role in tumor metastasis. As shown in Figure 3H, Rhy downregulated the expression of CXCR4, MMP-9, MMP-2 and proteins in HepG2 cells. 

### 2.10. Rhy Decreased the Phosphorylation Levels of Various Kinase Phosphorylation Sites in HepG2 Cells

**Figure 4:**
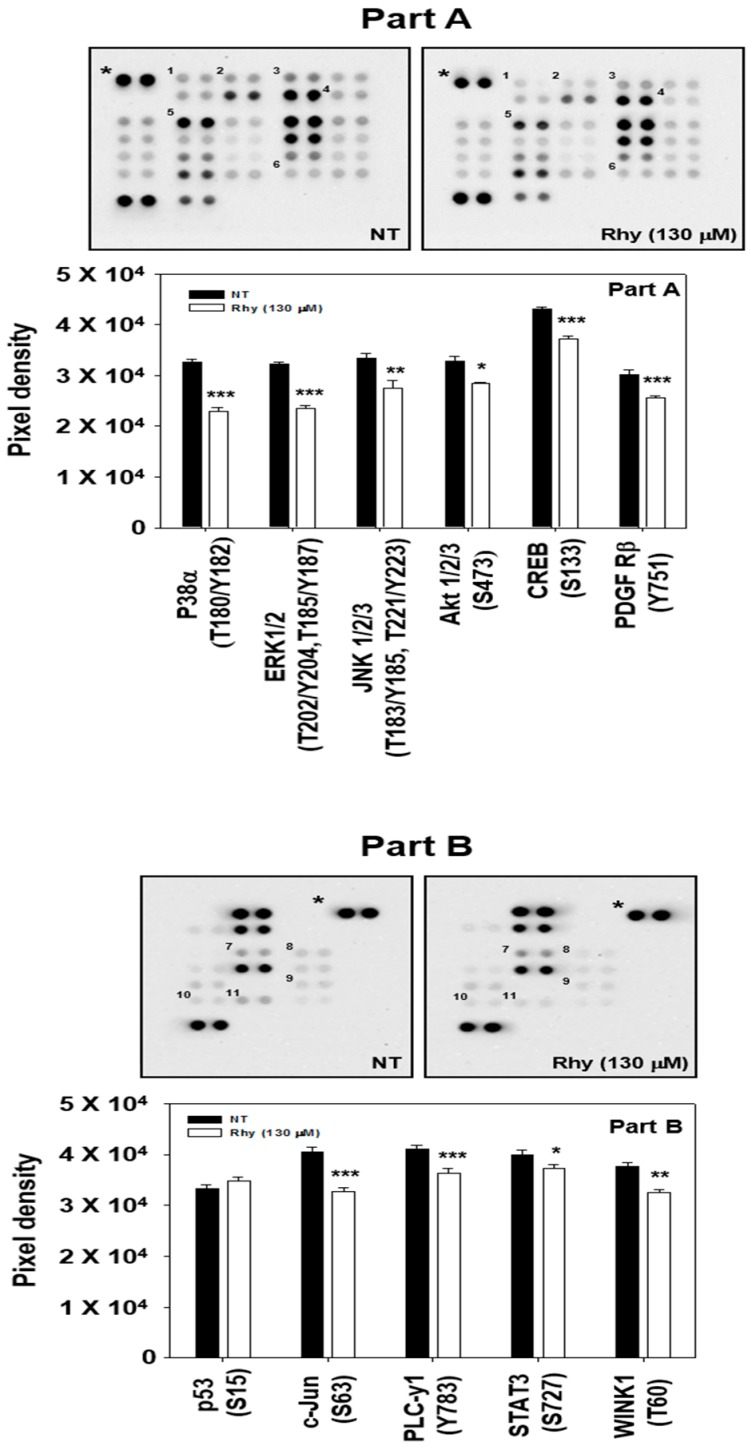
The Human Phospho-Kinase Array detects several phosphorylated proteins in Rhy-treated HepG2 cells. (**A**,**B**) HepG2 cells (1 × 10^7^ cells/well) were incubated with Rhy (130 µM) for 12 h. Parts A and B of the array were each incubated with 330 µg of cell lysate. Arrays were performed according to the manufacturer’s protocols using Human Phospho-Kinase Array Kit (R&D Systems, Minneapolis, MN, USA), and array images are shown. Array profiles created by quantifying the mean spot pixel densities are shown. Graphs represent spot intensities of indicated proteins. Values represent the mean ± SD of triplicate cultures (*** *p* < 0.001, ** *p* < 0.01, * *p* < 0.05).

To examine the effects of Rhy on intracellular signaling cascades, we screened the phosphorylation status of multiple cellular kinases in human HepG2, using the human phospho-kinase antibody array kit (Figure 4A,B). Incubation of these membranes with cell lysates can specifically indicate which kinases can be potentially activated in tumor cells and also as whether the same kinases can respond to the various pharmacological inhibitors. Activated p38, ERK, JNK, CREB, c-Jun, Akt, and STAT3 were strongly expressed in non-treated cells. Rhy treated cells showed a decrease in phosphorylated p38, ERK, JNK, CREB, c-Jun, Akt, and STAT3 levels. Phospho-p53 protein was found to be overexpressed upon Rhy treatment. Therefore, in the next experiments, we focused to determine the effect of Rhy on the p38, ERK, JNK, CREB, c-Jun, Akt, STAT3, and p53 signaling pathways in HepG2 cells.

### 2.11. Rhy Inhibited Phosphorylation of p38, ERK, JNK, CREB, c-Jun, Akt, STAT3, and Enhanced Phosphorylation of p53 in HepG2 Cells

**Figure 5:**
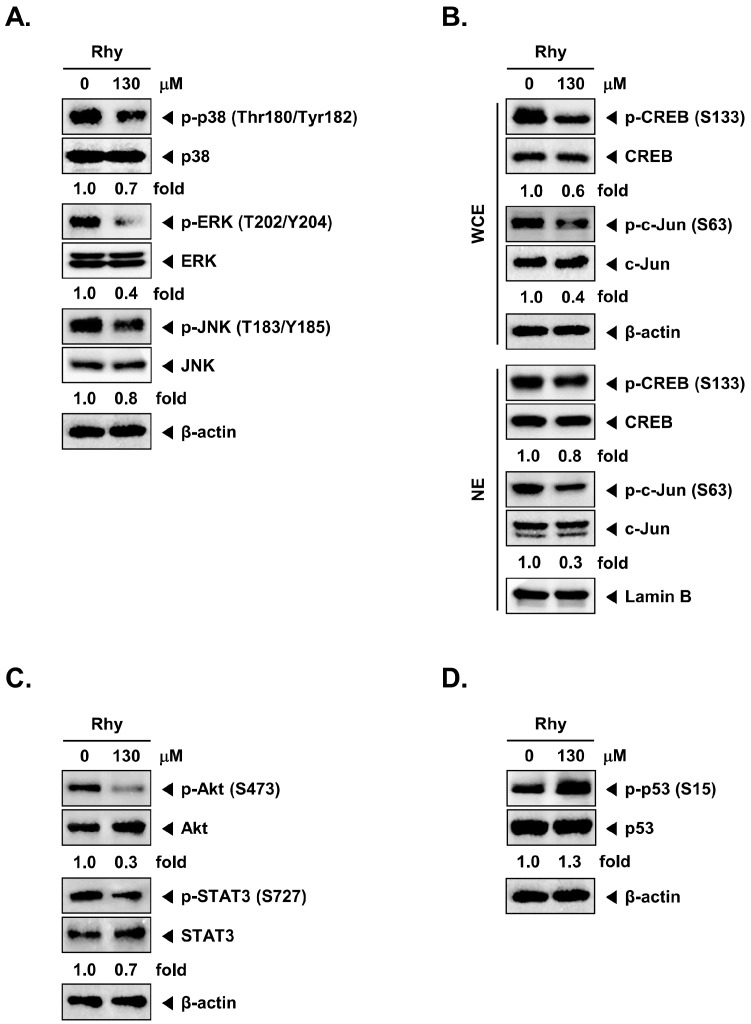
Effect of Rhy on various signal transduction pathways. (**A**) HepG2 cells (1 × 10^6^ cells/well) were incubated with Rhy (130 µM) for 12 h. Whole-cell extracts were prepared, and 20 µg of the whole-cell lysate was resolved by sodium dodecyl sulfate polyacrylamide gelelectrophoresis, electrotransferred to nitrocellulose membrane, sliced from the membrane based on the molecular weight, and then probed with antibodies against p-p38, p38, p-ERK, ERK, p-JNK, and JNK as described in Materials and Methods. The same blots were stripped and reprobed with β-actin antibody to verify equal protein loading. The results shown here are representative of three independent experiments; (**B**) HepG2 cells (1 × 10^6^ cells/well) were treated with Rhy (130 µM) for 12 h. After that, whole-cell extracts and nuclear proteins were extract were equal amounts of lysates were analyzed by Western blot analysis using antibodies against p-CREB, CREB, p-c-Jun, and c-Jun. The same blots were stripped and reprobed with β-actin and Lamin B antibody to verify equal protein loading. The results shown here are representative of three independent experiments; (**C**) HepG2 cells (1 × 10^6^ cells/well) were incubated with Rhy (130 µM) for 12 h. Whole-cell extracts were prepared, and 20 µg of the whole-cell lysate was resolved by sodium dodecyl sulfate polyacrylamide gelelectrophoresis, electrotransferred to nitrocellulose membrane, sliced from the membrane based on the molecular weight, and then probed with antibodies against p-Akt, Akt, p-STAT3, and STAT3 as described in Materials and Methods. The same blots were stripped and reprobed with β-actin antibody to verify equal protein loading. The results shown here are representative of three independent experiments; (**D**) HepG2 cells (1 × 10^6^ cells/well) were incubated with Rhy (130 µM) for 12 h. Whole-cell extracts were prepared, and 20 µg of the whole-cell lysate was resolved by sodium dodecyl sulfate polyacrylamide gelelectrophoresis, electrotransferred to nitrocellulose membrane, sliced from the membrane based on the molecular weight, and then probed with antibodies against p-p53, and p53 as described in Materials and Methods. The same blots were stripped and reprobed with β-actin antibody to verify equal protein loading.

Data obtained from our phospho-kinase antibody array studies were further confirmed by Western blotting analysis. Cells were treated with the indicated concentration of Rhy for 12 h. Rhy suppressed the phosphorylation of p38, ERK, JNK, CREB, c-Jun, Akt and STAT3, but enhanced the phosphorylation of p53 in HepG2 cells and had no effect on the expression of total p38, ERK, JNK, CREB, c-Jun, Akt, STAT3, p53 and β-actin proteins (Figure 5A–D).

## 3. Discussion

**Figure 6:**
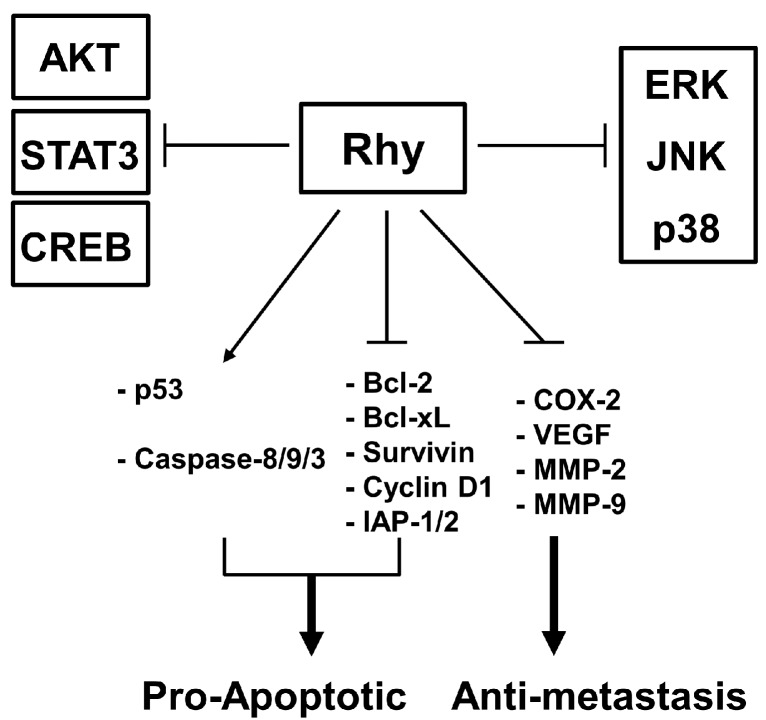
A schematic diagram depicting various molecular targets modulated upon Rhy treatment in tumor cells.

The aim of this study was to investigate the anticancer and anti-metastatic activities of Rhy against tumor cells. We found that Rhy exhibited highest cytotoxic activity against human hepatocellular carcinoma HepG2 cells. Therefore, we employed HepG2 cells to investigate the detailed mechanism of action of anticancer effects of Rhy. Interestingly, we found that Rhy downregulated the expression of various genes that suppress apoptosis, mediate proliferation, invasion, and angiogenesis. Rhy also induced the inhibition of proliferation, cell cycle arrest, and apoptosis. We further observed that Rhy inhibited the cell migration, invasion, and constitutive CXCR4, MMP-9, and MMP-2 expression in HepG2 cells. We also noted that Rhy suppressed the phosphorylation of p38, ERK, JNK, CREB, c-Jun, Akt, and STAT3 (signal transducer and activator of transcription 3) and also enhanced the phosphorylation of p53 at Ser15 residue (Figure 6).

Among genus *Uncaria*, *Uncaria rhynchophylla* is the less-investigated species for its anticancer activity. Recently, the aqueous extract of *Uncaria rhynchophylla* has been reported to be beneficial for the treatment of hypertension, cancer and neurological disorders [17,18,19,20]. In this study, we found for the first time that Rhy inhibited the growth of a wide variety of cancer cells. We examined the molecular mechanisms of cancer cell death induced by Rhy. We found that Rhy induced apoptosis through the activation of diverse caspases, including 8, 9, and 3, and caused PARP cleavage. Caspase-3 can be activated by mitochondrial or intrinsic pathway involving caspase-9 or an extrinsic pathway involving caspase-8 [21,22]. The results of the present study provide evidence to indicate that Rhy induced apoptosis is partly dependent on the activation of both caspase-8 and caspase-9. We also found that Rhy decreased the expression of anti-apoptotic proteins, Bcl-2, Bcl-xL, survivin, IAP-1, and IAP-2 and increased the expression of pro-apoptotic protein, Bax. Bcl-2 family proteins are localized in mitochondria and regulate caspase activation [23]. Both Bcl-2 and Bcl-xL are known to form heterodimers with another pro-apoptotic member of Bcl-2 family of protein, Bad, and thus can suppress apoptosis [24]. Bax can induce apoptosis through the release of cytochrome c and induce the activation of caspases [25]. Another apoptosis-regulatory protein family, IAPs, is considered to be an important target to modulate apoptotic cell death in many cancer cells. Inhibitors of apoptosis proteins family proteins regulate apoptosis by binding and inhibiting caspases [26,27]. A variety of prior studies have provided conclusive evidence(s) that elevation in Bcl-2 expression can cause resistance to chemotherapeutic drugs, while decrease in Bcl-2 expression can promote apoptotic responses to anticancer drugs [28]. Our results also indicate that the ectopic expression of Bcl-2 proteins can blocks Rhy-induced apoptosis and this was to be associated with the suppression of PARP cleavage. 

The downregulation of cell cycle promoting cyclin D1 expression and upregulation of cell cycle inhibitor p21 expression by Rhy correlated with the suppression in proliferation and accumulation of cells in the sub-G1 phase of the cell cycle. We also found that Rhy induced the downregulation of pro-angiogenic and metastatic factors COX-2 and VEGF. The CXCR4 chemokine receptor is expressed in a variety of cancers and has been linked to proliferation, invasion, and metastasis in many tumor cells, thus CXCR4 has been suggested as an important therapeutic target for inhibiting cancer metastasis [29]. Additionally, the invasion and metastasis of malignant cells has been found to be closely associated with the degradation of basement membranes and stromal extracellular matrix (ECM) [30]. The matrix metalloproteinases (MMPs), such as MMP-9 and MMP-2, play a critical role in cancer cell invasion and metastasis, which can degrade most components of the ECM [31,32,33,34,35]. We further found that the downregulation of constitutive CXCR4, MMP-9, and MMP-2 expression by Rhy directly correlated with its observed significant anti-invasive effects in the HepG2 cells. 

The neuroprotective effect of IRN has been reported to be associated with the enhancement of p-CREB expression mediated via the activation of PI3K/Akt/GSK-3*β* signaling pathways [36]. Rhy can also contribute to the regulation of MAPKs, NF-κB, STAT3, and Akt signal transduction in diverse disease models [5,36,37]. In the present study, using phospho-kinase array analysis, we have identified a few important potential targets for Rhy in HepG2 cells. Our studies offer first evidence about the ability of Rhy to suppress the phosphorylation of MAPK, Akt, STAT3 and to enhance the phosphorylation of p53. Mitogen-activated protein kinases (MAPKs) such as ERK, p38 kinase, and JNK pathways regulate cellular proliferation, apoptosis, and differentiation [38]. We show that Rhy inhibited the phosphorylation of ERK, p38, JNK(c-Jun) using both human phospho-antibody array system and Western blot analysis. However, it is still not entirely clear whether the diverse molecular targets modulated by Rhy to exhibit various neuroprotective effects may also play a prominent role in its observed anticancer effects in diverse tumor cell lines. 

Recent studies have reported that CREB is involved tumor initiation, progression and metastasis, thereby supporting its role as a proto-oncogene [39]. MAPKs (e.g., p38 and ERK) can phosphorylate and activate CREB-1, thereby promoting the pro-survival signaling that is important for cancer development and progression [40]. We also found that Rhy can substantially suppress the phosphorylation of CREB in HepG2 cells.

Signal transducer and activator of transcription 3 (STAT3) is an oncogenic transcription factor that is constitutively activated in many human cancers. STAT-3 can regulate the expression of genes that mediate survival, proliferation, invasion, and angiogenesis. Several natural chemopreventive agents have been found to be quite effective in suppressing STAT3 activation [41]. STAT3 activation is also tightly regulated by phosphorylation by intrinsic upstream tyrosine kinase and by MAPKs (e.g., p38 and ERK) [42,43,44]. The suppression of STAT3 phosphorylation by Rhy also correlated with the downregulation of STAT3-regulated gene products in HepG2 cells. These results suggest that STAT3 may be a possible target contributing to the anticancer effects of Rhy. 

Akt can regulate a number of downstream target genes which may have pleiotropic effects on both cell survival and proliferation [45], and the deregulation of Akt/p70S6K signal pathways can contribute significantly to the processes of tumorigenesis and metastasis [46,47]. In this study, remarkable reduction of Akt phosphorylation upon Rhy treatment could play a critical role in the inhibition of tumorigenesis. 

The p53 tumor suppressor gene is frequently found to be mutated in many human carcinomas [48]. Activation of p53 in response to a variety of stress signals can result in cell death. The role of p53 in apoptosis has been intensely studied for decades [49]. It is known that p53 phosphorylation at the Ser-15 residue is responsible for the activation of apoptotic machinery [50]. We observed that Rhy can increase the phosphorylation of p53 at Ser15 residue using both human phosphor-kinase array and Western blot analysis. The present study has few major limitations. First, most of the in vitro experiments have been predominantly performed in only one tumor cell line namely, HepG2. Second, preclinical therapeutic efficacy of Rhy has not been validated under in vivo settings. Lastly, no systematic analysis has been performed related to the toxicity as well as pharmacokinetic profile of Rhy. 

Overall, our results indicate that the anticancer and anti-metastatic effects of Rhy in HepG2 cells are mediated through the regulation of various signal transduction cascades and provide a strong rationale for further pursuing the use of Rhy for preclinical validation in appropriate tumor models. 

## 4. Materials and Methods

### 4.1. Reagents

Isorhynchophylline (Rhy) was dissolved in dimethyl sulfoxide as a 100 mM stock solution and stored at −20 °C and then diluted as needed in cell culture medium. The 3-(4,5-dimethylthiazol-2-yl)-2,5-diphenyltetrazolium bromide (MTT), Propidium iodide (PI), Tris base, glycine, NaCl, sodium dodecylsulfate (SDS), bovine serum albumin (BSA), Roswell Park Memoria lIn stitute medium (RPMI) 1640, Minimum Essential Medium (MEM), Dulbecco’s Modified Eagle’s Medium (DMEM), fetal bovine serum (FBS), were obtained from Thermo Fisher Scientific Inc. (Waltham, MA, USA). Annexin V was purchased from BD Biosciences (Palo Alto, CA, USA). Anti-CXCR4 was obtained from Abcam (Cambridge, MA, USA). Anti-Cyclin D1, anti-cleaved caspase-3, anti-cleaved caspase-8, anti-caspase-8, anti-cleaved caspase-9, anti-caspase-9, anti-p-ERK, anti-ERK, anti-p-p38, anti-p38, anti-p-JNK, anti-JNK, anti-p-Akt, anti-p-STAT3, anti-p-p53, anti-p-CREP , anti-CREB were purchased from Cell Signaling Technology (Beverly, MA, USA). Anti-Bcl-2, anti-Bcl-xl, anti-Survivin, anti-IAP-1, anti-IAP-2, anti-COX-2, anti-VEGF, anti-MMP-9, anti-MMP-2, anti-Bax, anti-p21, anti-caspase-3, anti-PARP, anti-Akt, anti-STAT3, anti-p53, anti-p-c-Jun, anti-c-Jun, anti-β-actin, and horseradish peroxidase (HRP)-conjugated secondary antibodies were obtained from Santa Cruz Biotechnology (Santa Cruz, CA, USA). Nitrocellulose membrane and nylon membrane were purchased from Pall Corporation (Washington, NY, USA).

### 4.2. Cell Lines

Human various tumor cells including HepG2 (Liver), A549 (Lung), BxPC-3 (Pancreas), Caki-1 (Kidney), RPMI-8226 (Blood), 786-O (Kidney), Du145 (Prostate), FaDu (Head and Neck), H1299 (Lung), MDA-MB-231 (Breast), U266 (Blood), H4 (Brain), U87MG (Brain), T98G (Brain), LN18 (Brain), and IM-PHFA (Brain) cells were obtained from the American Type Culture Collection (Manassas, VA, USA). HepG2, A549, BxPC-3, Caki-1, RPMI-8226, 786-O, Du145, H1299, MDA-MB-231, and U266 cells were cultured in RPMI 1640 medium containing 10% FBS, penicillin (100 units/mL), and streptomycin (100 µg/mL). FaDu cells were cultured in MEM containing 10% FBS, penicillin (100 units/mL), and streptomycin (100 µg/mL). H4, U87MG, T98G, LN18, and IM-PHFA cells were cultured in DMEM containing 10% FBS, penicillin (100 units/mL), and streptomycin (100 µg/mL).

### 4.3. MTT Assay

Cell viability was measured by an MTT assay to detect NADH-dependent dehydrogenase activity. Fifty microliters of MTT solution (2 mg/mL) in 1× phosphate-buffered saline (PBS) was directly added to the cells, which were then incubated for 2 h to allow MTT to metabolize to formazan. Absorbance was measured with an automated spectrophotometric plate reader at a wavelength of 570 nm. Cell viability was normalized as relative percentages in comparison with untreated controls.

### 4.4. Western Blot Analysis

After the cells were treated with the indicated concentrations of Rhy, the HepG2 cells were lysed and the total protein concentrations were determined by Bradford reagent (Bio-Rad, Hercules, CA, USA). Equal amounts of lysates resolved on sodium dodecyl-polyacrylamide gel electrophoresis (SDS-PAGE) were transferred to a nitrocellulose membrane, and the membrane was blocked with 1× TBS containing 0.1% Tween 20 and 5% skim milk at room temperature. After the blocking, the membranes were incubated overnight at 4 °C with the respective primary antibodies. The membranes were washed three times and incubated with diluted horseradish peroxidase (HRP)-conjugated secondary antibodies (1:5000) for 1 h at room temperature. After four times washing, the membranes were detected using an enhanced chemiluminescence (ECL) kit (Millipore, Bedford, MA, USA).

### 4.5. Transfection with pEGFP-Bcl-2 Plasmids

We used a commercially available electroporation system, the Neon™ Transfection System (Invitrogen, Carlsbad, CA, USA) for transfection experiments. HepG2 cells were prepared for transfection after cells were resuspended with 120 µL of Neon Resuspension Buffer R for every one million cells. For each electroporation, HepG2 cells with 0.25 µg of pEGFPBcl-2 plasmids (provided by Case Western Reserve University) were aliquoted into a sterile microcentrifuge tube. After 48 h of transfection, HepG2 cells were treated with 130 µM of Rhy for 48 h, and whole-cell extracts were prepared for Bcl-2, PARP, and β-actin analysis by Western blot.

### 4.6. Cell Cycle Analysis

To determine apoptosis, cell cycle analysis was performed using PI. After treatment with 130 µM of Rhy for 36 h, the cells were collected, washed with cold PBS, fixed with 70% ethanol, and incubated for 30 min at 37 °C with 0.1% RNase A in PBS. Cells were then washed, resuspended, and stained in PBS containing 10 µg/mL of PI for 30 min at room temperature. Cell distribution across the cell cycle was analyzed with a flow cytometry (Becton–Dickinson, Heidelberg, Germany). Acquisition and analysis of the data were performed using Cell Quest 3.0 software (BD Biosciences, Becton–Dickinson, Franklin Lakes, NJ, USA).

### 4.7. Annexin V Assay

One of the early indicators of apoptosis is the rapid translocation and accumulation of the membrane phospholipid phosphatidylserine from the cell’s cytoplasmic interface to the extracellular surface. This loss of membrane asymmetry can be detected using the binding properties of Annexin V. To detect apoptosis, we used Annexin V antibody conjugated with the fluorescent dye fluorescein isothiocyanate (FITC). HepG2 cells were treated with 130 µM of Rhy for 36 h and then subjected to Annexin V and PI staining. The cells were washed and observed accordingly with a flow cytometry (Becton–Dickinson). Acquisition and analysis of the data were performed using Cell Quest 3.0 software.

### 4.8. TUNEL Assay

Late apoptotic cell death was determined using a Roche Diagnosis TUNEL (terminal transferase mediated dUTP-fluorescein nick end labeling) assay kit according to the manufacturer’s instructions. Briefly, HepG2 cells treated with 130 µM of Rhy for 36 h and which were washed with cold PBS. The cells were seeded after being fixed with 4% paraformaldehyde for 30 min and washed twice with PBS for 2 min. The resuspended cells were put in a permeabilization solution (0.1% Triton X-100 and 0.1% Sodium citrate) at 4 °C for 20 min, and then the cells were washed with cold PBS. The cells in 45 µL of TUNEL enzyme and TUNEL label mixture were incubated for 1 h at 37 °C in a humidified atmosphere in the dark. After being washed with PBS, cells were analyzed by a flow cytometery (FACScan Calibur, BD Biosciences, Becton–Dickinson). Acquisition and analysis of the data were performed using Cell Quest 3.0 software.

### 4.9. Real-Time Cell Proliferation Analysis

HepG2 cells (5000 cells/well) were seeded onto 16-well E-plates, integrated with gold microelectrode arrays, then incubated in real-time cell analysis (RTCA) was carried out with the xCELLigence System (Roche, Mannheim, Germany). Application of a low-voltage alternating current signal generates an electric field between the electrodes, which is modulated by the cells covering the electrodes. Cell proliferation in the wells results in changes in the impedance readout, obtained from each well with the RTCA DP Instrument. The generated signal is displayed in arbitrary units, referred to as the cell index. After initial incubation on the E-plates for 18 h, HepG2 cells were treated with the Rhy (65 and 130 µM). Non-treated samples were used as controls. The cell index was monitored for 48 h, with measurements every 15 min.

### 4.10. Live and Dead Assay

To measure apoptosis, we used the Live and Dead assay (Invitrogen, Carlsbad, CA, USA), which determines intracellular esterase activity and plasma membrane integrity. This assay employs calcein, a polyanionic dye, which is retained within the live cells and provides green fluorescence. It also employs the ethidium monomer dye (red fluorescence), which can enter the cells only through damaged membranes and bind to nucleic acids but is excluded by the intact plasma membrane of live cells. Briefly, HepG2 cells were seeded at a density of 3 × 10^4^ cells/well in 8-well slide chamber. The cells were incubated with 130 µM of Rhy for 36 h. Cells are stained with the Live and Dead reagent (5 µM ethidium homodimer, 5 µM calcein-AM) and then incubated at 37 °C for 30 min. Cells were analyzed under an Olympus FluoViewFV1000 confocal microscope (Olympus, Tokyo, Japan).

### 4.11. Wound Healing Assay

Before plating the cells, two parallel lines were drawn at the underside of the wells to serve as fiducial marks demarcating the wound areas to be analyzed. Before inflicting the wound, the cells should be fully confluent. The growth medium was aspirated off and replaced by calcium-free phosphate buffered saline to prevent killing of the cells at the edge of the wound by exposure to high calcium concentrations before two parallel scratch wounds were made perpendicular to the marker lines with a sterile 200 µL automated pipette tip. Thereafter, the calcium-free medium was then changed to medium with or without 65 µM of Rhy. After 60 h, the wounds were observed using bright field microscopy, and multiple images were taken at areas flanking the intersections of the wound and the marker lines at the start and end of the experiment. Gap distance of the wound was measured at three different sites using Photoshop software, and the data were normalized to the average of the control. Graphs were plotted against the percentage of migration distance the cells moved before and after treatment, normalized to control.

### 4.12. Invasion Assay

We employed the Roche xCELLigence Real-Time Cell Analyzer (RTCA) DP instrument (Roche Diagnostics GmbH) to measure cellular invasion. The RTCA DP instrument uses the cellular invasion/migration (CIM)-Plate 16, which features microelectronic sensors integrated onto the underside of the microporous polyethylene terephthalate membrane of a Boyden-like chamber. For invasion experiments, the top chamber of the CIM-Plate 16 was coated with Matrigel (BD Biosciences, San Diego, CA, USA) before addition of the medium to the bottom chamber. The CIM-Plate 16 was assembled by placing the top chamber onto the bottom chamber and snapping the two together. Serum-free medium was placed in the top chamber to hydrate and preincubate the membrane for 1 h in the CO_2_ incubator at 37 °C before obtaining a background measurement. Cells were lightly trypsinized, pelleted, and resuspended at the indicated cell densities in serum-freemedium. Once the CIM-Plate 16 has been equilibrated, it was placed in the RTCA DP station, and the background cell-index values were measured. The CIM-Plate 16 was then removed from the RTCA DP station, and the cells are added to the top chamber at the desired density.

### 4.13. Human Phospho-Kinase Array

For antibody arrays, 300 µg of cellular extracts were incubated with the Human Phospho-Kinase Array Kit (Proteome Profiler™; R&D Systems) following manufacturer’s instructions. Densitometry values for antibody array experiments were estimated by the ImageJ 1.6.0 software (National Institutes of Health, Bethesda, MA, USA) and were expressed as arbitrary units. Multiple film exposures were used to verify the linearity of the samples analyzed and to avoid saturation of the film. In antibody arrays, the average signal of the pair of duplicate spots, representing each phosphorylated kinase protein, was calculated after subtraction of background values (pixel density) from negative control spots and normalization to average values from positive control spots.

### 4.14. Statistical Analysis

All numeric values are represented as the mean ± SD. Statistical significance of the data compared with the untreated control was determined using the Student unpaired *t*-test. Significance was set at *p* < 0.05.
